# Capzb2 Interacts with β-Tubulin to Regulate Growth Cone Morphology and Neurite Outgrowth

**DOI:** 10.1371/journal.pbio.1000208

**Published:** 2009-10-06

**Authors:** David A. Davis, Meredith H. Wilson, Jodel Giraud, Zhigang Xie, Huang-Chun Tseng, Cheryl England, Haya Herscovitz, Li-Huei Tsai, Ivana Delalle

**Affiliations:** 1 Department of Pathology and Laboratory Medicine, Boston University School of Medicine, Boston, Massachusetts, United States of America; 2 Howard Hughes Medical Institute, Massachusetts Institute of Technology, Cambridge, Massachusetts, United States of America; 3 Picower Institute for Learning and Memory, Riken-MIT Neuroscience Research Center, Department of Brain and Cognitive Sciences, Massachusetts Institute of Technology, Cambridge, Massachusetts, United States of America; 4 Department of Neurosurgery, Boston University School of Medicine, Boston, Massachusetts, United States of America; 5 Department of Physiology & Biophysics, Boston University School of Medicine, Boston, Massachusetts, United States of America; Adolf-Butenandt-Institut, Germany

## Abstract

Capping protein (CP) is a heterodimer that regulates actin assembly by binding to the barbed end of F-actin. In cultured nonneuronal cells, each CP subunit plays a critical role in the organization and dynamics of lamellipodia and filopodia. Mutations in either α or β CP subunit result in retinal degeneration in *Drosophila*. However, the function of CP subunits in mammalian neurons remains unclear. Here, we investigate the role of the β CP subunit expressed in the brain, Capzb2, in growth cone morphology and neurite outgrowth. We found that silencing Capzb2 in hippocampal neurons resulted in short neurites and misshapen growth cones in which microtubules overgrew into the periphery and completely overlapped with F-actin. In searching for the mechanisms underlying these cytoskeletal abnormalities, we identified β-tubulin as a novel binding partner of Capzb2 and demonstrated that Capzb2 decreases the rate and the extent of tubulin polymerization in vitro. We mapped the region of Capzb2 that was required for the subunit to interact with β-tubulin and inhibit microtubule polymerization. A mutant Capzb2 lacking this region was able to bind F-actin and form a CP heterodimer with α2-subunit. However, this mutant was unable to rescue the growth cone and neurite outgrowth phenotypes caused by Capzb2 knockdown. Together, these data suggest that Capzb2 plays an important role in growth cone formation and neurite outgrowth and that the underlying mechanism may involve direct interaction between Capzb2 and microtubules.

## Abstract 2

An actin regulatory protein unexpectedly also controls microtubule polymerization during the formation and maintenance of cellular outgrowths in neurons.

## Author Summary

Neuronal growth, migration, and survival depend on the regulated formation of cellular outgrowths called neurites. Extension of normal neurites requires coordinated interactions between cytoskeletal networks made up of microfilaments (composed of F-actin) and microtubules (formed by tubulin) in structures called growth cones that form at the tips of growing neurites. Capping protein (CP) is a heterodimer that regulates F-actin assembly in a variety of cell types. Surprisingly, the neuronal CP β subunit, Capzb2, not only regulates F-actin assembly, but also inhibits microtubule polymerization by direct interaction with tubulin. We further show that this function of Capzb2 is required for establishment of the normal shape of growth cones and the appropriate length of neurites. Our data thus reveal an unexpected, dual role for CP in the regulation of both microfilaments and microtubules in neurons.

## Introduction

Growth cone morphology and neurite outgrowth are controlled by temporally and spatially coordinated interactions between two major cytoskeletal networks, F-actin and microtubules [1]–[3]. Recapitulation of these developmental cytoskeletal interactions is required for a successful regenerative response and plasticity following the injury of adult neurons [4]–[6]. On the other hand, cytoskeletal abnormalities characterize neurodegenerative diseases such as Alzheimer disease [7]–[10], Parkinson disease [7]–[9],[11], amyotrophic lateral sclerosis [7]–[9],[12],[13], and Huntington disease [7]–[9],[14]. A recent study using a *Drosophila* model of tauopathy suggests that abnormalities in the actin cyoskeleton play a causative role in neurotoxicity [15]. In support of this, a mutagenesis screen in *Drosophila* revealed that homozygous mutations in either of the two subunits of F-actin capping protein (CP) resulted in the accumulation of F-actin and degeneration of photoreceptors [16].

CP is an F-actin binding protein that functions as an α/β heterodimer. The heterodimer binds the barbed end of F-actin, thereby blocking the access of actin monomers to the fast growing end of F-actin. This binding is mediated by the two extreme C-terminal regions of the α- and β-subunits [17],[18]. In *Drosophila*, each CP unit is encoded by a single gene. In mammals, the α-subunit is encoded by three separate genes [19], whereas the β-subunit is encoded by one gene that gives rise to three isoforms [20]. One of these isoforms, Capzb2, is predominantly expressed in the brain [20]. Both *Drosophila* and mammalian CP subunits have been shown to play a critical role in the organization and dynamics of lamellipodia and filopodia in nonneuronal cells by regulating the actin cytoskeleton [21],[22]. However, the role of the CP subunits in mammalian neurons is currently unknown.

In this study, we investigate the function of Capzb2 in growth cone formation and neurite outgrowth, processes that are relevant to the regenerative response of injured neurons [4]–[6]. We provide evidence that Capzb2 plays an essential role in these processes and that, surprisingly, the underlying mechanism may involve direct interaction between Capzb2 and microtubules.

## Results

### Capzb2 RNA Interference Causes Shorter Neurites in Hippocampal Neurons

**Figure 1:**
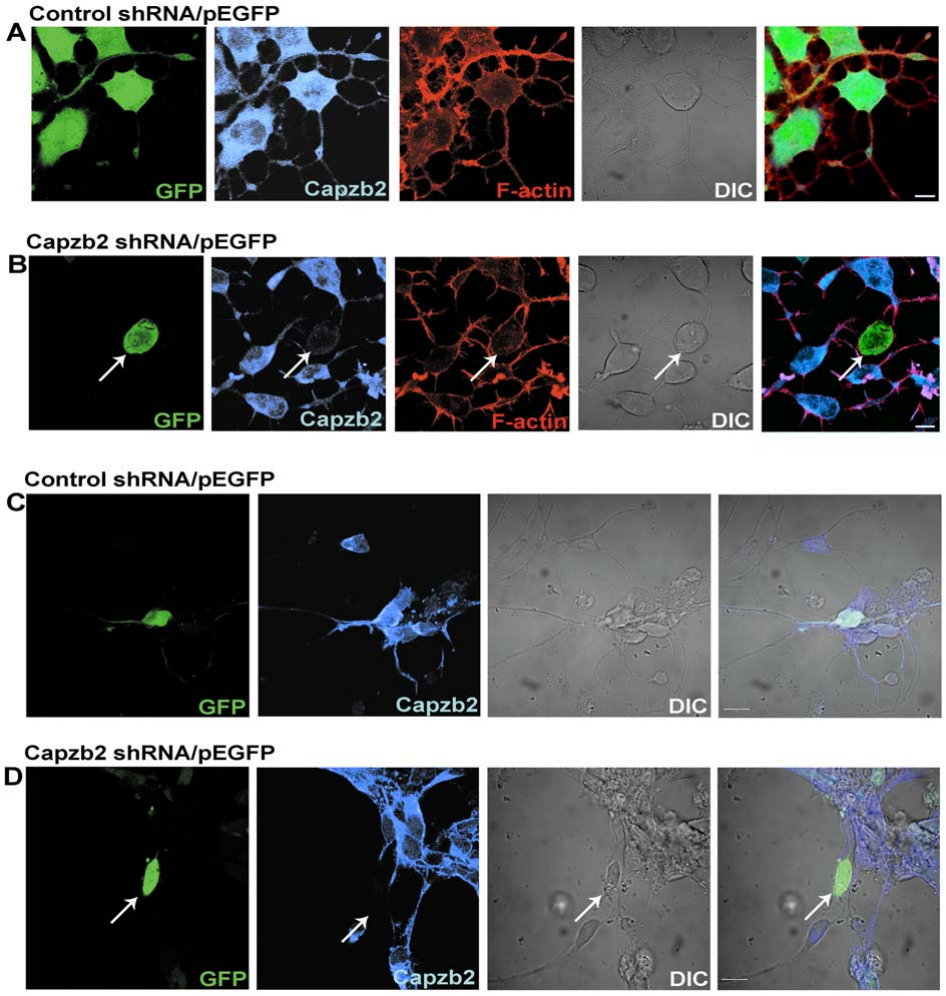
Capzb2 shRNA efficiently knocks down the expression of Capzb2 in CAD cells and primary hippocampal neurons. CAD (mouse neuroblastoma) cells (A and B) and embryonic rat hippocampal neurons (E17) (C and D) were transfected with either control shRNA (A and C) or Capzb2 shRNA (B and D) and fixed 72 h posttransfection. In all, green represents cells transfected with either control or Capzb2 shRNA/pEGFP. Capzb2 signal (blue) is detected in CAD cells and hippocampal neurons transfected with control shRNA/pEGFP and in nontransfected cells (A and C), whereas it is greatly reduced in cells transfected with Capzb2 shRNA/pEGFP (arrows in [B and D]). Actin labeling (red) (B) and the DIC image (B and D) highlight the absence of processes in Capzb2 shRNA/pEGFP-transfected cell. Scale bars indicate 10 µm.

To investigate the neuronal function of Capzb2, we generated a small hairpin RNA construct (Capzb2 shRNA). Capzb2 shRNA effectively knocked down the expression of Capzb2 in mouse neuroblastoma CAD cells and embryonic rat hippocampal neurons (Figure 1A–1D and Figure S1).

We next compared the levels of expression of Capzb2 protein in the embryonic and adult mouse hippocampus and cortex. Capzb2 was detected in both the cortex and hippocampus of embryonic and adult mice. However, the signal was much stronger in the adult hippocampus than in the adult cortex (Figure S2A). Compared to the embryonic stage, the expression of Capzb2 in the adult was significantly reduced in the cortex, but not in the hippocampus (Figure S2B).

**Figure 2:**
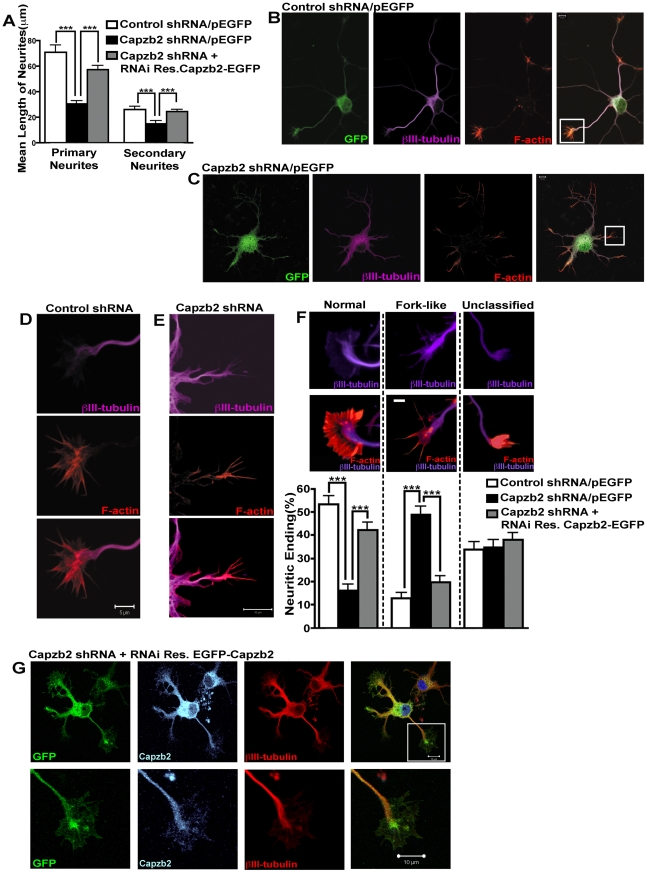
Capzb2 RNAi causes shorter neurites and alters morphology of growth cones/actin-rich neuritic endings in hippocampal neurons. (A) Mean length of primary and secondary neurites of neurons transfected with control shRNA/pEGFP, Capzb2 shRNA/pEGFP, or Capzb2 shRNA/RNAi resistant (Res) Capzb2-EGFP (ratio 4∶1) were analyzed 72 h posttransfection and 4% paraformaldehyde fixation. Capzb2 shRNA transfection results in reduced length of both primary and secondary neurites; concomitant transfection of RNAi Res Capzb2-EGFP reverses the effect of Capzb2 RNAi. Mean values ±s.e.m. (*n* = 100 neurons in each category) are depicted; *** = *p*<0.001. (B and C) Representative shRNA/pEGFP transfected neurons: (B) control shRNA/pEGFP and (C) Capzb2 shRNA/pEGFP; GFP (green), F-actin (red), and βIII-tubulin (purple); scale bar indicates 10 µm. (D) Higher magnification of a normal growth cone delineated by the white box in (B), characterized by an F-actin–rich periphery (red) and a βIII-tubulin–rich C-zone (purple); scale bar indicates 5 µm. (E) Higher magnification of a growth cone delineated by the white box in (C) in which there is overlap of F-actin (red) and βIII-tubulin (purple) signal; scale bar indicates 5 µm. Note the altered morphology as characterized by the absence of lamellipodia, resulting in a fork-like appearance. (F) Representative images of βIII-tubulin (purple) and F-actin (red) in normal, fork-like, and unclassified growth cones/actin-rich neuritic endings (scale bar indicates 5 µm), and quantification of each of these categories in neurons transfected with control shRNA/pEGFP, Capzb2 shRNA/pEGFP, and Capzb2 shRNA+RNAi Res Capzb2-EGFP (rescue transfection). The neurons were fixed in PHEM buffer 48 h posttransfection. The neuritic ending morphology classified as fork-like lacked lamellipodia, and the βIII-tubulin signal (purple) extended into the periphery and overlapped with F-actin (red) rather than being confined centrally as in normal growth cones. Fork-like “growth cones” dominated in neurons transfected with Capzb2 shRNA, whereas normal growth cones were significantly more common in control and rescue conditions. The third category, unclassified, represents neuritic endings that could not be classified as either normal or fork-like. These neuritic endings had uniform appearance and were found in similar frequencies across the experimental conditions. more than 230 growth cones/actin-rich neuritic endings from neurons belonging to each category were analyzed; mean values ±s.e.m. are depicted; *** = *p*<0.001. (G) EGFP-Capzb2 is present in the neuronal soma and processes including the growth cones where it colocalizes with βIII-tubulin. The lower panels are higher magnifications of the area delineated by the white box in the upper right-hand panel.

Because Capzb2 RNA interference (RNAi) seemed to reduce the length of processes in CAD cells and primary neurons (Figure 1), we decided to quantify the effect of Capzb2 shRNA on mouse hippocampal neurites. We measured the lengths of all of the neurites of GFP-immunoreactive neurons that were transfected with one the following combination of constructs: control shRNA/pEGFP, Capzb2 shRNA/pEGFP, or Capzb2 shRNA+RNAi-resistant Capzb2-EGFP. The measurement of GFP-positive neurites was performed on the images of these neurons obtained with bright-field settings and the appropriate differential interference contrast (DIC) to ensure the complete outline of all of the neuronal processes. The analysis showed that Capzb2 shRNA significantly reduced the length of both primary and secondary neurites (Figure 2A). This effect of Capzb2 shRNA was abolished by the expression of RNAi-resistant Capzb2-EGFP (Figure 2A). These data suggest that Capzb2 plays a critical role in neurite outgrowth of hippocampal neurons.

### Capzb2 RNAi Alters Growth Cone Morphology in Hippocampal Neurons

The morphology of growth cones is mainly dependent on two cytoskeletal proteins, F-actin and tubulin. To investigate whether Capzb2 might regulate the cytoskeleton in growth cones of cultured hippocampal neurons, we examined the effect of Capzb2 shRNA on the distribution of F-actin and tubulin in growth cones of transfected hippocampal neurons. In control neurons, the majority of growth cones exhibited normal morphology, comprised of a central tubulin-rich zone and a peripheral F-actin–rich zone (Figure 2B, 2D, and 2F). However, in Capzb2 shRNA-transfected neurons, growth cones more often exhibited an abnormal “fork-like” morphology, characterized by the absence of lamellipodia and the extension of microtubules into the peripheral F-actin–rich zone (Figure 2C, 2E, and 2F). We quantified the number of normal growth cones and abnormal fork-like ones in neurons transfected with control shRNA, Capzb2 shRNA, or both Capzb2 shRNA and RNAi-resistant EGFP-Capzb2 (Figure 2F). Normal growth cones were the dominant form of neuritic endings in both control shRNA-transfected neurons and neurons cotransfected with Capzb2 shRNA and RNAi-resistant EGFP-Capzb2 (Figure 2G), whereas fork-like structures were mostly associated with neurons transfected with Capzb2 shRNA only. Neuritic endings that could not be clearly classified as either normal growth cones or fork-like structures were designated as “unclassified.” The percentage of neuritic endings that belonged to the unclassified category was the same among all of the groups.

**Figure 3:**
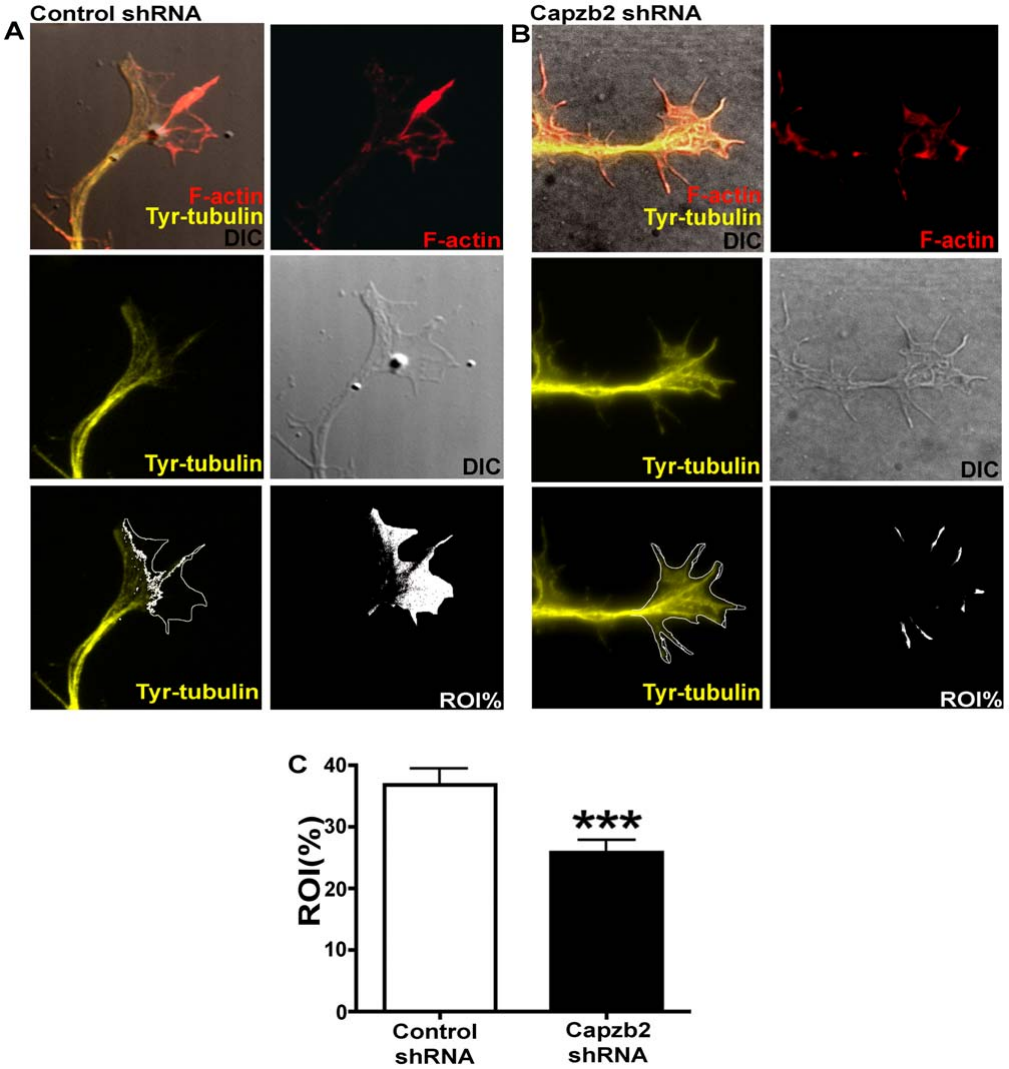
Comparison of growth cone area not invaded by microtubules in neurons transfected with either control shRNA/pEGFP or Capzb2 shRNA/pEGFP. (A and B) Visualization of microtubules in the growth cones was obtained with tyrosinated α-tubulin antibody signal (Tyr-tubulin, yellow); F-actin (red) upon CytD treatment. The percentage of growth cone area not invaded by microtubules (ROI%, white area) was obtained upon subtraction of Tyr-tubulin signal from the total growth cone area visualized on DIC image. The image left of the panels labeled ROI% shows the ROI border (white line overlay on Tyr-tubulin signal image) composed of the line indicating microtubule most distal position (based on Tyr-tubulin signal) and the outline of the growth cone (based on DIC image). (C) The average ROI% was significantly lower in neurons transfected with Capzb2 shRNA (*n* = 63, blinded analysis from three experiments) in comparison to controls (*n* = 85, blinded analysis from three experiments). Mean values ±s.e.m. are depicted; *** = *p*<0.001.

These results suggest that Capzb2 influences microtubule extension into the peripheral domain of a growth cone, raising the question whether Capzb2 may act directly on microtubules or indirectly via actin cytoskeleton [23]. To address this question, we assessed the effect of Capzb2 on microtubules in neurons treated with cytochalasin D (CytD), which removes the actin meshwork and thus prevents inhibitory action of actin retrograde flow on microtubules in growth cones. In a blinded image analysis, we quantified the area of the growth cone not invaded by microtubules in neurons transfected with control shRNA or Capzb2 shRNA and treated with CytD (Figure 3). To visualize microtubules in the growth cones, we used tyrosinated α-tubulin antibody because the tyrosinated form of microtubules is the dominant one in the growth cones [24]. The average percentage of growth cone area not invaded by microtubules (percentage of region of interest, ROI%) was significantly lower in neurons transfected with Capzb2 shRNA than in control (Figure 3C). These data suggest that Capzb2 may directly influence microtubule extension into the peripheral domain of a growth cone.

### Capzb2 Associates with βIII-Tubulin and Decreases Tubulin Polymerization In Vitro in a Concentration-Dependent Manner

**Figure 4:**
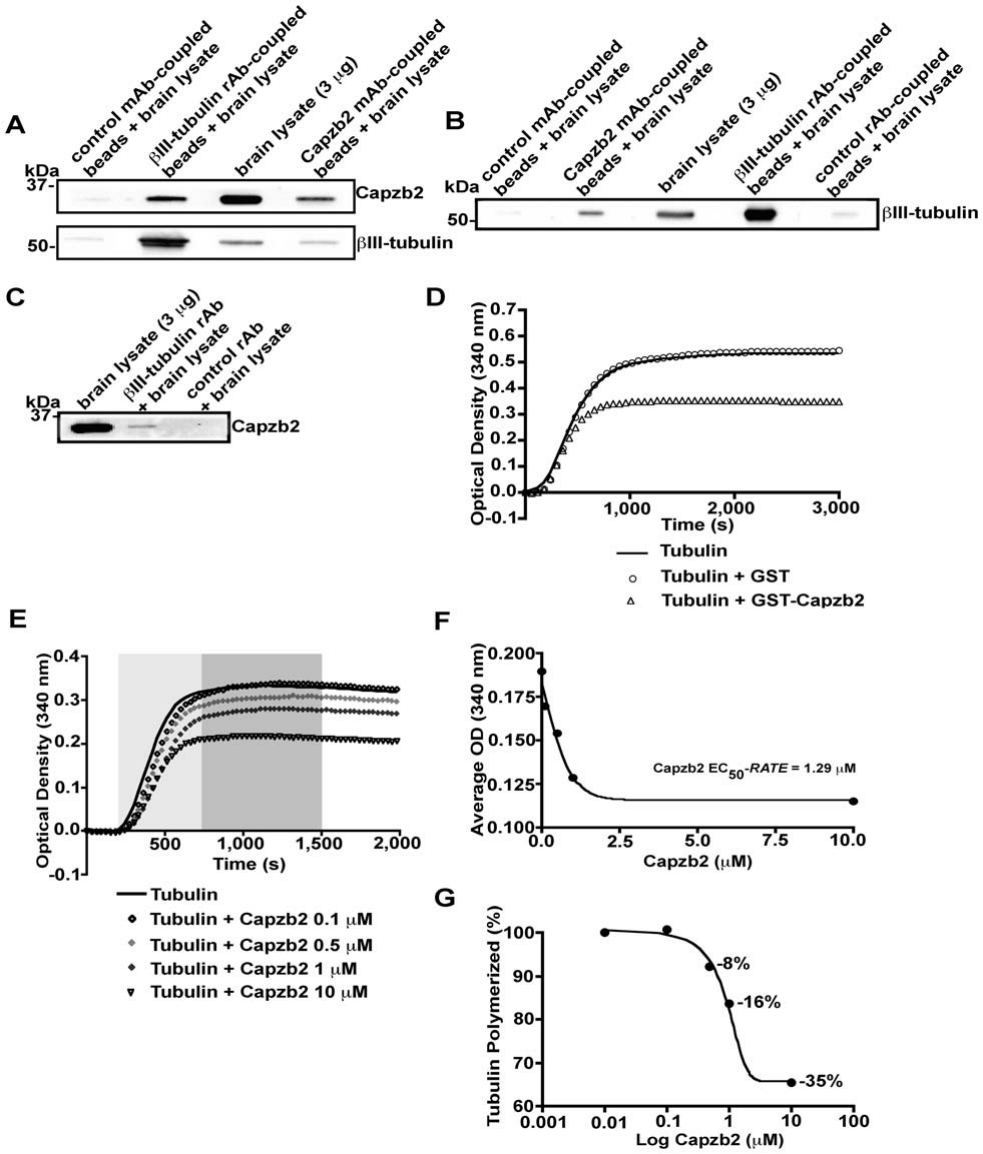
Capzb2 coimmunoprecipitates with βIII-tubulin in brain lysates and decreases tubulin polymerization in vitro. (A) In order to avoid overlap of the heavy chain signal (∼50 kDa) with the βIII-tubulin signal (∼55 kDa) in the immunoprecipitation using the Capzb2 monoclonal antibody (mAb), the antibody was coupled to the protein G/A beads through cross-linking. βIII-tubulin and Capzb2 signals were each detected following immunoprecipitation from brain lysate with both βIII-tubulin rabbit antibody (rAb)-coupled beads and Capzb2 mAb-coupled beads. The expected molecular weight of Capzb2 (28–32 kDa) and βIII-tubulin (∼55 kDa) is confirmed in brain lysate (3 µg). (B) Similarly, βIII-tubulin signal was detected specifically both in immunoprecipitations with Capzb2 mAb-coupled beads and with βIII-tubulin rAb-coupled beads, as confirmed in the brain lysate lane. (C) Capzb2 was also specifically precipitated also by βIII-tubulin rAb that was not cross-linked to the protein G/A beads; the expected molecular weight of Capzb2 was detected in the brain lysate lane. (D) Tubulin monomers were incubated in the presence of 0.05 mg/ml tau only or together with 10 µM of GST-Capzb2 or 10 µM of GST-tag. GST-tag alone did not alter tubulin polymerization. GST-Capzb2 decreased the maximal level of microtubule assembly by approximately 40%. Curves represent mean change in optical density (OD) at 30-s intervals. (E) Capzb2 decreases the rate and the extent of tubulin polymerization in a concentration-dependent manner. The lightly shaded region indicates the exponential phase of tubulin polymerization. The darkly shaded region indicates the interval in which the final extent of tubulin polymerization is achieved. (F) To determine the concentration of Capzb2 required for 50% reduction in the rate of tubulin polymerization (Capzb2 EC_50_-*RATE*), a nonlinear sigmoidal curve was fitted to the average OD during the exponential phase of tubulin polymerization (interval 180–750 s; lightly shaded in [E]) versus Capzb2 concentration. Each point depicted represents the average of 20 OD readings obtained for each Capzb2 concentration examined (0, 0.1, 0.5, 1, and 10 µM) during the interval 180–750 s; *R* ^2^ (correlation coefficient) = 0.99. (G) In the presence of increasing of Capzb2 concentrations of 0.5, 1, and 10 µM, the extent of tubulin polymerization is reduced by 8%, 16%, and 35%, respectively. The average of 25 OD readings obtained for each of the Capzb2 concentrations examined was normalized against the average OD reading in the absence of Capzb2 during 750–1,500-s interval (darkly shaded in [E]) in which the final extent of tubulin polymerization is achieved for a given reaction. Capzb2 concentration is displayed on a log of 10 scale, so the first depicted point (tubulin polymerization in the absence of Capzb2; OD value = 100%) on the obtained nonlinear sigmoidal curve (*R* ^2^ = 0.99) corresponds to 0.01 on the Log Capzb2 (µM). The concentration of 0.1 µM Capzb2 (the second depicted point on the curve) does not reduce the extent of tubulin polymerization although it does affect the rate (as seen in [F]).

It has previously been hypothesized that proteins that interact with both F-actin and microtubules may play a role in growth cone dynamics, such as regulating the invasion of microtubules [1]. Interestingly, in addition to actin, β-tubulin was among the few proteins that were pulled down from mouse brain lysates by GST-Capzb2 (unpublished data). The microtubule invasion phenotype caused by Capzb2 shRNA (Figures 2E, 2F, and 3B) further raised the possibility that Capzb2 might interact with neuronal microtubules. In support of this possibility, we found that Capzb2 and βIII-tubulin coimmunoprecipitate from brain lysates (Figure 4A–4C).

To further explore the association between Capzb2 and tubulin, we tested the ability of Capzb2 to affect the formation of microtubules in absorbance-based tubulin polymerization assays. Using conditions that promote tubulin assembly [25], we examined whether purified GST-tagged Capzb2 (Figure S3) might alter the degree of tubulin polymerization. Although the GST tag (Figure S3) alone did not alter tubulin polymerization, GST-Capzb2 decreased the maximal level of microtubule assembly (Figure 4D). In addition, the effect of Capzb2 on the rate and the extent of tubulin polymerization is dependent on the concentration of Capzb2 (Figure 4E–4G).

### Capzb2 Lacking the Microtubule-Interacting Region Fails to Rescue the Capzb2 RNAi Phenotype

**Figure 5:**
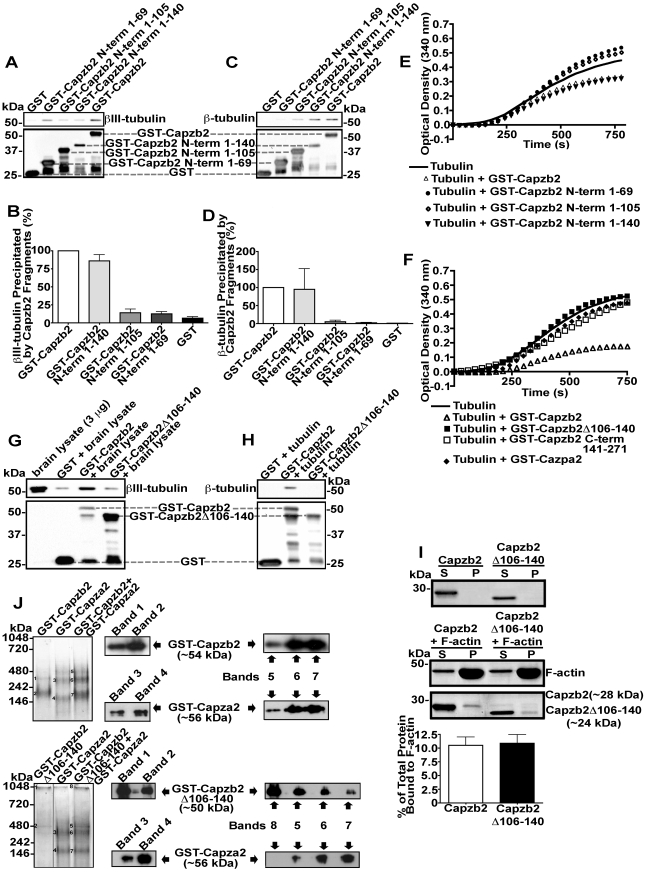
Identification of the Capzb2 region necessary for binding tubulin and for the effect on tubulin polymerization. (A–D) Specific GST-tagged Capzb2 protein segments show differential ability to pull down βIII-tubulin from mouse brain lysates (A and B) or to bind β-tubulin in vitro (C and D). The average ratio (*n* = 3) between the signal for the GST-tagged Capzb2 full-length/protein segment and the βIII-tubulin signal obtained in pull-down assays from brain lysates (C) or the β-tubulin signal in in vitro binding assays (D) is shown. Note that GST-tagged full-length Capzb2 and GST-tagged Capzb2 N-terminus 1–140 show a strong affinity for β-tubulin, whereas smaller GST-Capzb2 fragments (N-terminus 1–105 and N-terminus 1–69) showed progressively weaker affinities for β-tubulin in vitro; GST-tag alone serves as a negative control. (E) Tubulin polymerization assay was performed as in Figure 4D, in the presence of 10 µM GST-Capzb2, or one of three progressively shorter GST-Capzb2 N-terminus segments. Similar to GST-Capzb2, GST-Capzb2 N-terminus 1–140 decreased the rate and extent of polymerization. However, GST-Capzb2 N-terminus 1–105 and GST-Capzb2 N-terminus 1–69 did not have a negative effect on tubulin polymerization. (F) Tubulin polymerization assay was performed as in Figures 4D and 5E, in the presence of 10 µM GST-Capzb2, 10 µM GST-Capzb2Δ106–140, 10 µM GST-Capzb2 C-terminus 141–271, or 10 µM GST-Capza2. Capzb2 deletion mutant lacking region Δ106–140 did not have a negative effect on tubulin polymerization. In addition, neither Capzb2 C-terminus 141–271 nor the other CP unit, Capza2, affected tubulin polymerization, whereas Capzb2 decreased both the rate and the extent of polymerization. (G and H) When compared with GST-Capzb2, GST-Capzb2Δ106–140 could not specifically precipitate βIII-tubulin from brain lysate (G) and was not able to bind β-tubulin in vitro (H); GST tag alone served as a negative control (G and H). (I) Capzb2 or Capzb2Δ106–140 were incubated alone or in the presence of F-actin. Upon centrifugation, both the supernatant (S) and pellet (P) of each reaction were analyzed by SDS-PAGE followed by Coomassie Brilliant Blue staining. A representative experiment of four performed is displayed. In the presence of F-actin, both Capzb2Δ106–140 and Capzb2 are comparably cosedimented with F-actin. Densitometry indicates that the percentage of Capzb2Δ106–140 (*n* = 7) and the percentage of Capzb2 (*n* = 8) bound to F-actin do not differ significantly (*p* = 0.69). (J) GST-Capzb2+GST-Capza2 and GST-Capzb2Δ106–140 (1 µM)+GST-Capza2 (1 µM) were incubated for 10 min at 4°C and subjected to native PAGE. Gels were analyzed with Denville Blue stain. Following destaining, the bands were excised and their content was extracted under alkaline conditions and subjected to Western blot analysis. Note that the GST-Capzb2Δ106–140 signal is weak due to the relatively lower affinity of this mutant for the Denville Blue stain in comparison to GST-Capza2. Thus, the GST-Capzb2Δ106–140 lane is overexposed. Western blot analysis indicates the identity of GST-Capzb2, GST-Capza2, and GST-Capzb2Δ106–140. When incubated with GST-Capza2, both GST-Capzb2 and GST-Capzb2Δ106–140 give rise to a similar profile of heterodimer complexes.

To identify specific region(s) in the Capzb2 sequence necessary for the interaction with β-tubulin, we expressed and purified truncated GST-tagged Capzb2 proteins (see Figure S3). GST-tagged full-length Capzb2 and GST-tagged Capzb2 N-terminus 1–140 exhibited a similar ability to pull down βIII-tubulin from brain lysates (Figure 5A and 5B) and bind tubulin β-subunits in vitro (Figure 5C and 5D), whereas shorter GST-tagged Capzb2 segments (N-termini 1–105 and 1–69) were inefficient (Figure 5A–5D). To identify the region of the Capzb2 sequence indispensable for inhibiting tubulin polymerization, microtubule assembly assays were performed with these same GST-tagged protein segments (Figure 5E). Consistent with the pull-down and in vitro binding experiments, GST-tagged full-length Capzb2 and GST-tagged Capzb2 N-terminus 1–140, but not the shorter GST-tagged segments, inhibited tubulin polymerization.

On the basis of these data, we concluded that Capzb2 residues 106–140 might be important for Capzb2 interaction with microtubules. We also tested Capzb2 C-terminus 141–271 and Capza2, the other CP subunit with secondary and tertiary structures resembling those of Capzb2 [26]. Neither of these proteins had an effect on tubulin polymerization (Figure 5F and Figure S3). As predicted, the deletion mutant Capzb2Δ106–140 did not affect microtubule polymerization in vitro (Figure 5F and Figure S3). Furthermore, Capzb2Δ106–140 was unable to either precipitate or bind β-tubulin (Figure 5G and 5H), although it was able to bind F-actin (Figure 5I and Figure S5) and form a CP heterodimer with α2-subunit (Capza2) (Figure 5J).

**Figure 6:**
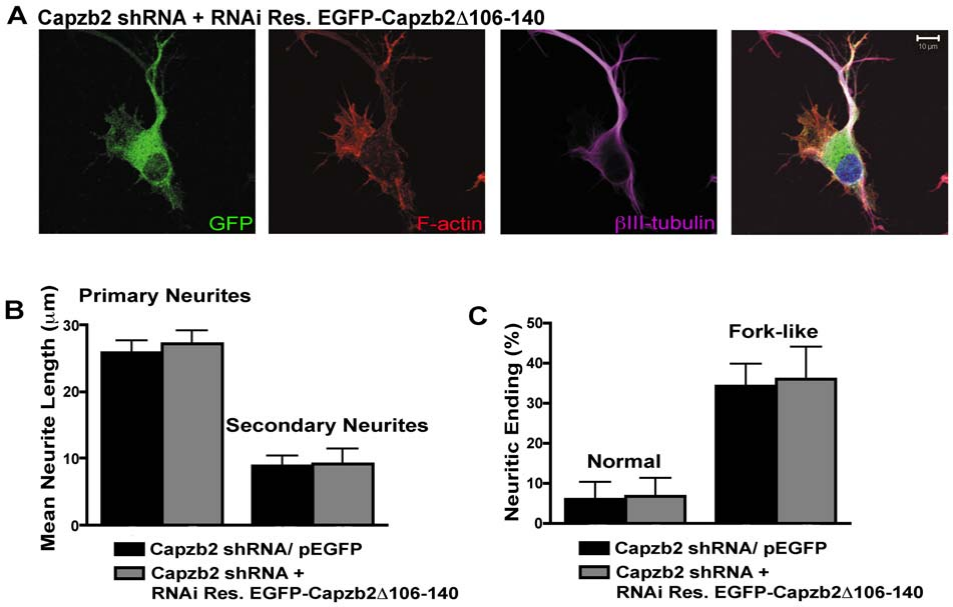
Capzb2 lacking the region that interacts with microtubules fails to rescue the Capzb2 RNAi phenotype. (A) Neurons were analyzed following PHEM fixation 48 h posttransfection with either Capzb2shRNA/pEGFP or Capzb2 shRNA+EGFP-Capzb2Δ106–140; a representative neuron in shown: GFP channel indicates GFP-Capzb2Δ106–140 distribution similar to the distribution of GFP-Capzb2 signal shown in Figure 2G; however, no growth cones are seen ([A] green: GFP, red: F-actin, purple: βIII-tubulin, and blue: nucleus); scale bar indicates 10 µm. (B) No significant differences (*p*>0.05) in the lengths of primary and secondary neurites were observed between neurons transfected with Capzb2 shRNA/pEGFP and neurons transfected with Capzb2 shRNA+EGFP-Capzb2Δ106–140; mean values ±s.e.m. are depicted (*n* = 25). (C) No significant differences (*p*>0.05) in percentages of normal and fork-like growth cones/neuritic endings were observed between neurons transfected with Capzb2 shRNA/pEGFP (25 neurons = 67 growth cones/actin-rich neuritic endings) and neurons transfected with Capzb2 shRNA+EGFP-Capzb2Δ106–140 (25 neurons = 50 growth cones/actin-rich neuritic endings); mean values ±s.e.m. are depicted.

We next examined whether Capzb2Δ106–140 could rescue the neurite outgrowth and growth cone phenotypes caused by Capzb2 shRNA. To this end, we created EGFP-tagged Capzb2Δ106–140, which was well expressed in mammalian cells (Figure S4). Nevertheless, the frequency of neurons transfected with Capzb2 shRNA+EGFP-Capzb2Δ106–140 (Figure 6) was lower than that in neurons transfected with Capzb2 shRNA/pEGFP or Capzb2 shRNA+RNAi-resistant Capzb2-EGFP (see Figure 2F). We found that neuritic lengths of primary and secondary neurites transfected with Capzb2 shRNA+EGFP-Capzb2Δ106–140 (representative neuron in Figure 6A) were similar to those in neurons transfected with Capzb2 shRNA/pEGFP (Figure 6B). Few normal growth cones were found in either group examined, whereas fork-like neuritic endings were common in both populations (Figure 6C). These data suggest that the ability of Capzb2 to regulate growth cone formation and neurite outgrowth is dependent on its microtubule-interacting region.

## Discussion

CP is an F-actin binding protein that plays a key role in actin assembly. Previous studies have shown that CP regulates the organization and dynamics of lamellipodia and filopodia in nonneuronal cells and supports the survival of *Drosophila* photoreceptors. In this study, we investigated the function of Capzb2, the CP β-subunit isoform expressed in the brain. We have shown that Capzb2 is essential for neurite outgrowth and growth cone morphology in cultured mammalian neurons. In addition, we have uncovered a new role for Capzb2 in cytoskeletal regulation: inhibiting microtubule polymerization by direct interaction with tubulin.

The organization and dynamics of F-actin and microtubules play a central role in neurite initiation and extension. Thus, as an important regulator of F-actin assembly, Capzb2 is likely critical for neurite outgrowth. Consistent with this, silencing of Capzb2 in these neurons by shRNA results in short neurites (Figure 2). This defect was rescued by an RNAi-resistant vector for the expression of Capzb2-EGFP (Figure 2A). Together, these data suggest that Capzb2 is essential for neurite outgrowth.

The length of individual neurites is largely dependent on a functional growth cone with well-described dynamic morphology [1],[2],[27],[28]. The temporal and spatial changes in the organization of the actin and tubulin cytoskeleton in a growth cone have been extensively studied [1],[2],[28],[29]. In general, two well-defined zones have been observed: a tubulin-rich central zone (C-zone) and an F-actin–rich peripheral zone (P-zone). The latter is composed of filopodial spikes separated by the lamellipodia, relatively splayed structures supported by a branched F-actin network. Central and peripheral zones are separated by the transitional zone (T-zone), which in addition to being rich in F-actin, also contains a few microtubules on their route to the P-zone. Within the P-zone, these microtubules preferentially grow along the filopodia, and not within lamellipodia [1],[29]. The growth cones displaying characteristic zonal organization were rare in Capzb2 shRNA-transfected neurons in comparison to controls. Instead, the predominantly observed structure associated with neuritic endings in Capzb2 shRNA transfected neurons did not contain lamellipodia, supporting the notion that CP β-subunit is a necessary component for CP function in the formation of lamellipodia [21],[22]. Additionally, the βIII-tubulin signal, rather than being confined to a central tubulin-rich zone characteristic of a normal growth cone, colocalized with F-actin signal in the periphery of a fork-like neuritic ending. These data suggest that Capzb2 plays a critical role in the functional morphology of a growth cone, thereby promoting neurite outgrowth.

Based on previous studies in nonneuronal cells [21],[22],[26], the regulation of F-actin may underlie the function of Capzb2 in growth cone formation. Our data, however, suggest that a direct interaction between Capzb2 and microtubules may also be involved. We demonstrated the association between Capzb2 and β-tubulin by coimmunoprecipitation, GST pull-down, and in vitro binding assays (Figures 4A–4C and 5A–5H). Furthermore, we showed that a mutant Capzb2 lacking the microtubule-interacting region, although capable of binding F-actin and forming a CP heterodimer (Figure 5I and 5J), failed to rescue the defects in neurite outgrowth and growth cone formation (Figure 6). Finally, in the absence of branched F-actin filament meshwork, microtubule distribution in the growth cone is dependent on the presence of Capzb2 (Figure 3). Together, our data suggest that the interaction between Capzb2 and microtubules may contribute to the function of Capzb2 in growth cone formation and neurite outgrowth. Interestingly, the interaction between CapZ and β-tubulin has been uncovered in mass spectrometry screen for the alterations in protein-target binding in vivo in response to spatial learning [30].

**Figure 7:**
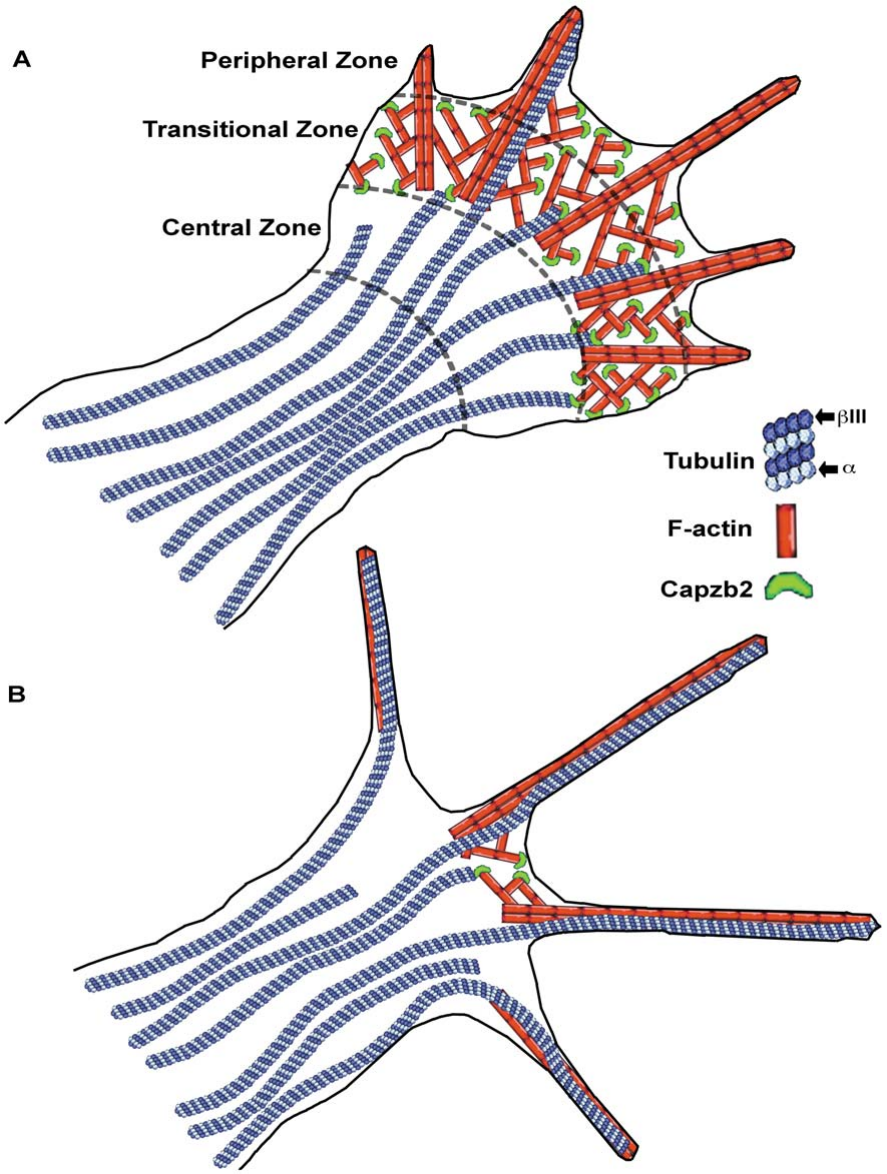
Model of the actin/Capzb2/microtubule interaction in the growth cone. (A) In the normal growth cone, Capzb2 in the lamellipodia interacts with microtubules to decrease tubulin polymerization. Note that microtubules are concentrated in the C-zone; only occasionally do microtubules traverse into the T-zone to grow along the filopodia in the P-zone. (B) In the absence of an interaction with Capzb2, growth of microtubules is unrestrained, resulting in the complete overlap between microtubules and the F-actin–rich periphery. Note that lamellipodia are absent.

In the context of the established cytoskeletal organization in the growth cone [1] and our data, we propose the following model for the function of Capzb2 effect in the neuronal growth cone: Capzb2 decreases the rate and the extent of tubulin polymerization within the T-zone and lamellipodia, thereby restricting the majority of microtubules to the C-zone and allowing only a few microtubules to enter the filopodia (Figure 7A). Without the interaction with Capzb2, the growth of microtubules into the periphery of the growth cone is unrestrained. Consequently, microtubules invade all of the filopodia, reaching their distal tips. This results in a complete overlap of microtubules and F-actin in the periphery and the disappearance of the lamellipodia (Figure 7B). Thus, the normal growth cone structure characterized by the distinct C-, T-, and P-zones is replaced by fork-like neuritic endings in which the tubulin and F-actin signals completely overlap. The premature disappearance of the growth cone results in the premature termination of neurite growth and thus diminished neurite length.

Our study, together with the known function of CP in actin assembly, suggests that Capzb2 may act as a key coordinator of the assembly of F-actin and microtubules, the two major cytoskeletal networks that determine the morphology and dynamics of growth cones. The importance of Capzb2 for neurite outgrowth is particularly interesting in light of the recently established role of Ena/VASP in neuritogenesis in vivo [31]. In contrast to the role that CP plays in supporting the formation of lamellipodia, Ena/VASP are F-actin barbed-end capping proteins that promote the formation of filopodia [31]. Thus, it is likely that coordinated assembly of lamellipodia and filopodia, controlled by proteins with opposing activity, is essential for growth cone formation and neurite outgrowth.

## Materials and Methods

### Generation of Capzb2 shRNA

An siRNA construct builder program (Ambion) was used to generate 55–60-mer oligonucleotides encoding 21-mer hairpin sequences specific to the Capzb2 mRNA target, with 3′ single-stranded overhangs for ligation into p*Silencer* 2.0 under promoter U6 (Ambion). To test the efficiency of the generated RNAi sequences, CAD cells (mouse neuroblastoma cells) were transfected with the four generated Capzb2 shRNA sequences as well as with the control shRNA, a random sequence without homology to any known mRNA, using Lipofectamine 2000 (Invitrogen). The sequence *tt gct gga gtg atc ctc ata a* effectively reduced the expression of Capzb2 48 h posttransfection (see Figure S1).

### Mouse Brain and CAD Cell Lysate Preparation and Western Blots

Total protein extracts of whole brain, cortex, or hippocampus from Swiss Webster adult mice and embryonic day 16 (E16) embryos were prepared in ELB buffer (pH 7.5) (10 mM EDTA [pH 8.0], 300 mM NaCl, 100 mM TRIS [pH 7.5], 0.2% v/v NP40). Total protein extracts from CAD cells were prepared in RIPA buffer (150 mM NaCl, 50 mM Tris [pH 8.0], 10% v/v SDS, 0.5% v/v DOC, 1% v/v NP-40). Protein concentrations were determined by BCA assay (Sigma-Aldrich) using BSA as standards. Proteins were fractionated on SDS-PAGE and transferred to PVDF membrane for Western blot analysis. Membranes were incubated with anti-Capzb2 1∶1,000 (DSHB mAb 3F2.3), anti-GAPDH 1∶4,000 (Ambion), anti-tubulin 1∶5,000 (Sigma), or anti-GST 1∶4,000 (Santa Cruz Biotechnology), followed by incubation with appropriate secondary HRP-conjugated antibodies (Santa Cruz Biotechnology). Proteins were visualized using Image Station 440CF (Kodak Digital Science) or by the Enhanced Chemiluminescence System (Pierce). Quantification of signal was performed using ImageJ software version 1.37v (National Institutes of Health).

### Electroporation and Primary Culture of Mouse Hippocampal Neurons

Pregnant Swiss Webster dams were euthanized in a CO_2_ chamber, followed by cervical dislocation on E16; the fetuses were subsequently removed and developing hippocampi were dissected in 1 mM HBSS (Invitrogen)+20 mM HEPES (pH 7.3) followed by dissociation at 37°C with 2.5% trypsin+1% DNase. An Amaxa Biosystem apparatus was used according to the manufacturer's instructions to deliver the following combinations of DNA constructs into the dissociated hippocampal neurons: Capzb2 shRNA+pEGFP, control shRNA+pEGFP, Capzb2 shRNA+RNAi-resistant Capzb2-EGFP, and Capzb2 shRNA+RNAi-resistant EGFP-Capzb2Δ106–140 (a construct coding for a deletion of amino acids 106–140). The mouse Capzb2 cDNA was a gift from Dorothy Schafer, University of Virginia. Capzb2 full-length and mutant sequence Capzb2Δ106–140 were obtained by PCR (using primers 5′-caa ggg tac ctc aac act gct gct ttc tct tca agg c-3′ and 5′-cgg aat tct atg agc gat cag cag ctg gac tgc-3′ from GST-Capzb2Δ106–140 template—see below) and cloned into pEGFP-C1 vectors (Clontech) for expression in mammalian cells. We have tested Capzb2 constructs carrying EGFP in either the C- or N-terminus. For the full-length Capzb2 construct, the site of EGFP did not seem to influence the frequency or the brightness of GFP-immunoreactive neurons. However, the deletional mutants with an EGFP-tag (in addition to Capzb2Δ106–140, we also made Capzb2Δ70–105-EGFP, a mutant that had very low expression in neurons) carrying EGFP at the N-terminus were more reliably detectable in comparison to those with an EGFP at the C-terminus. All electroporation procedures were performed with 4×10^6^ cells and 2.4 µg of shRNA plus 0.6 µg of pEGFP vector/construct (4∶1 ratio).

### Immunofluorescence of Primary Neurons and Quantitative Analyses

Forty-eight or 72 h following transfection, cells were fixed in PHEM buffer (60 mM PIPES, 25 mM HEPES, 2 mM MgCl_2_, 10 mM EGTA, 120 mM sucrose [pH 7.4]) prewarmed to 37°C or in 4% v/v paraformaldehyde. Primary neurons were immunofluorescently labeled using the following primary antibodies: anti-Capzb2 (1∶50 dilution, DSHB, mAb 3F2.3), anti–βIII-tubulin (1∶200 dilution, Sigma), anti-tyrosinated α-tubulin (1∶2,500 dilution, Chemicon), and anti-GFP (1∶500 dilution, Aves Labs), followed by labeling with Alexa-fluor secondary antibodies (1∶500 dilution, Molecular Probes). F-actin was labeled with rhodamine phalloidin (1∶50 dilution, Molecular Probes). For the documentation and quantification of phenotypic alterations in transfected neurons (length of primary and secondary neurites, growth cone morphology, growth cone area free of microtubule invasion after CytD treatment, 0.4 µM for 10 min at 37°C), a Zeiss or Olympus confocal microscope coupled with IPLab 4.01 software (BD Biosciences) was used. For quantitative analyses, confocal images of the transfected (GFP-positive) neurons were obtained with the 40× and/or 60× objectives. In addition to fluorescence images, using bright-field settings with the appropriate DIC prism, we obtained images documenting the complete outline of the neuron, its neurites, and growth cones/neuritic endings. All quantitative analyses were performed using the image analysis package included in the IPLab software. Primary neurites were measured from the soma of the neuron to the terminal end of the primary process. Secondary neurites were measured from the junction of the primary and secondary neurite to the terminal end of the secondary process. For the analysis of growth cone morphology, images of the F-actin and tubulin signals were also obtained, overlaid, and the actin-rich neuritic endings were classified as discussed in Results. For the analysis of growth cone area free of microtubule invasion after CytD treatment, an IPLab program was used to delineate a growth cone area on a DIC image (ROI). Following the subtraction of the area occupied by tyrosinated tubulin signal, the percentage of ROI (ROI%) free of tyrosinated tubulin signal was obtained.

### Immunoprecipitation

Adult mouse brain lysate (3 mg, 2 mg/ml ELB buffer) were incubated with the following antibodies: monoclonal Capzb2 (DSHB mAb 3F2.3, 1.6 µg), rabbit affinity isolated βIII-tubulin (10 µg, Sigma), control mouse (anti-GST, 1 µg, Santa Cruz Biotechnology), and control rabbit (anti-HA, 1 µg, Sigma) for 2 h at 4°C. Protein G/A beads (20 µl, Pierce) were washed three times in ELB buffer and incubated for 30 min at 4°C. Upon incubation with the brain lysates, beads were washed three times in ELB buffer, resuspended in 2× Laemmli buffer, and analyzed by Western blot.

### Tubulin In Vitro Polymerization Assay

Absorbance-based tubulin polymerization assays were performed to evaluate the effect of Capzb2 on the assembly of microtubules. Polymerization of bovine tubulin monomers (cytoskeleton, 60 µM) in general tubulin buffer (PEM: 80 mM PIPES, 2 mM MgCl_2_, and 1 mM EGTA [pH 6.9]) supplemented with 1 mM GTP and 10% v/v glycerol was initiated by incubation at 37°C, and changes in turbidity were measured by absorbance at 30-s intervals at 340 nm in a Powerwave x-1 microplate reader (BioTek). Duplicate samples were examined in each assay, and results shown are mean values for each condition. Tubulin was incubated in the presence of tau (0.8 µM) only or with added Capzb2 using the following concentrations: 0.1, 0.5, 1, and 10 µM. At the end of a polymerization assay, microtubules were negatively stained with 3% uranyl acetate and placed on grids for transmission electron microscopy to verify microtubule formation.

### Generation of GST-Tagged Capzb2 Protein Fragments and Constructs

The following glutathione S-transferase (GST)-tagged Capzb2 fragments were created by cloning the corresponding nucleotide sequences into a pGEX-4T-2 vector (GE Healthcare Life Sciences): Capzb2 (full-length Capzb2, 1–271), Capzb2 N-terminus 1–140, Capzb2 N-terminus 1–105, and Capzb2 N-terminus 1–69. Primers used to clone the corresponding Capzb2 fragments are as follows: Capzb2 (full-length) forward 5′-tcc tcc ccc ggg agc gat cag cag ctg gac t-3′, Capzb2 (full-length) reverse 5′-aag gaa aaa act cga gtc aac act gct gct ttc tct t-3′, Capzb2 N-terminus 1–140 forward 5′-cgg gat cca gcg atc agc agc tgg-3′, Capzb2 N-terminus 1–140 reverse 5′-cgg aat tct cat cca tct cca gct ttc-3′, Capzb2 N- terminus 1–105 forward 5′-ggc caa caa tgc ctt cga cta ata cc gaga c-3′, Capzb2 N-terminus 1–105 reverse 5′-gtc tcg gta tta gtc gaa ggc att gtt ggc c-3′, Capzb2 N-terminus 1–69 forward 5′-caa cag aga cgg gga ctg ata tag gtc acc g-3′, Capzb2 N-terminus 1–69 reverse 5′-cgg tga cct ata tca gtc ccc gtc tct gtt g-3′, Capzb2 C-terminus 141–271 forward 5′-cgg gat cct cca aga aga tca aag gct gc-3′, and Capzb2 C-terminus reverse 5′-cgg aat tct caa cac tgc tgc ttt c-3′. In order to create Capzb2Δ106–140 sequence, we performed fusion PCR reaction using primers 5′-tcc tcc ccc ggg agc gat cag cag ctg gac t -3′ and 5′-aag gaa aaa act cga gtc aac act gct gct ttc tct t-3′, whereas the template was a mixture of two PCR products. In creating each of the products, Capzb2 full-length sequence was used as a template: PCR product 1 was obtained using primers 5′-tcc tcc ccc ggg agc gat cag cag ctg gac t-3′ and 5′-ccc agc agc ctt tga tct tct tgg agt cga agg cat tgt tgg -3′; PCR product 2 was obtained using primers 5′-tcc aag aag atc aaa ggc tgc tgg-3′ and 5′-aag gaa aaa act cga gtc aac act gct gct ttc tct t-3′. Capza2 sequence was obtained from E17 mouse cDNA using primers 5′-cgt tag atc tat ggc gga tct gga gga gca g-3′ and 5′-atg aat tct tat gca ttc tgc atc tct ttg c-3′. The PCR products obtained were ligated into a pGEX-4T-2 vector, and the constructs were used to transform BL21-DE3 *E. coli* competent cells (Stratagene) or ArcticExpress (DE3)RP competent cells (Stratagene); protein production was induced using a 1 mM concentration of dioxane-free IPTG (Calbiochem). Following the protein production, bacterial cell pellets were prepared and resuspended in TNT buffer (50 mM TRIS [pH 8.0], 20 mM NaCl, 2.5 mM EDTA [pH 8.0], 0.5% v/v Tween-20, 5% v/v glycerol) supplemented with 1.5 mM DTT (dithiothreitol), 1 mM PMSF (phenylmethanesulphonylfluoride), and protease inhibitors (Complete Mini, EDTA-free protease inhibitor tablets, Roche). Bacterial cell lysates were prepared by sonication on ice followed by supplementation with Triton X-100 to a final concentration of 1% and centrifugation at 12,000 rpm at 4°C. The supernatant was collected and incubated with gluthathione sepharose beads (GE Healthcare Life Sciences) at 4°C followed by the elution of GST-tagged Capzb2 protein (full length and segments) from GST beads with the appropriate buffer (200 mM TRIS [pH 8.0], 0.1% v/v Triton X-100, 15 mM glutathione, 1 mM PMSF, and protease inhibitors, Roche) in Poly-Prep Chromatography Columns (Bio-Rad). The eluted proteins were dialyzed using a Dialysis Kit (Amersham Biosciences) against PBS overnight at 4°C and concentrated with Amicon Ultra (Millipore) centrifuge tubes according to the manufacturer's protocol. The final concentration of each protein fragment was determined by Bio-Rad protein assay (Bio-Rad).

### Thrombin Cleavage for GST-Capzb2 and GST-Capb2Δ106–140 Proteins

Thrombin protease (Amersham Biosciences) was used to remove the GST tag from the generated fusion proteins GST-Capzb2 and GST-Capzb2Δ106–140. Briefly, GST-Capzb2 and GST-Capzb2Δ106–140 were incubated with thrombin protease for 20 h at 4°C with rocking. Following the cleavage of GST, thrombin protease was removed using benzamidine sepharose beads (GE Healthcare). A Bradford assay was performed to determine the untagged proteins' concentrations.

### Tubulin In Vitro Binding Assay and Pull-Down from Brain Lysate

In vitro binding reactions between tubulin (cytoskeleton, 1, 2, or 3 µg) and each of the purified GST-Capzb2 segments or full-length protein (1, 2, or 3 µg) were carried out in binding buffer (200 mM NaCl, 0.2% v/v Triton X-100, 0.2 mg/ml BSA, and 50 mM TRIS [pH 7.5]) for 3 h at 4°C; in the experiment shown in Figure 5H, 3 µg of tubulin and 3 µg of Capzb2 full-length and deletion mutant were used. Alternatively, 3 µg of GST-Capzb2 segments or full-length protein or deletion mutant were incubated with brain lysate (3 mg, 2 mg/ml ELB buffer) for 3 h at 4°C. Capzb2 segments or full-length protein or deletion mutant were subsequently precipitated using 100 µl of 10% gluthathione sepharose beads in binding buffer (for in vitro binding assay) or 20 µl of gluthathione sepharose beads washed in ELB buffer (for pull-downs from brain lysate) that were previously added to the respective incubations for 1 h at 4°C. The precipitate was washed three times in the appropriate buffer and resuspended in 2× Laemmli buffer and subjected to Western blotting.

### Native Gel Electrophoresis

Equal amounts (1 µM) of GST-Capzb2 and GST-Capzb2 Δ106–140 were incubated with 1 µM of Capza2 (10 min, 4°C, rocking). Upon adding the native sample buffer (2×; 60 mM Tris HCl, 50% v/v glycerol, and 2% w/v Bromophenol Blue), the samples were loaded on a 4%–20% Tris-glycine gel (Invitrogen) and subjected to electrophoresis (150 min, 125 V, Surelock apparatus, Invitrogen). Gels were stained with Denville Blue according to manufacturer's instructions and destained in deionized water. Protein bands were excised using a clean scalpel and homogenized in gel extraction buffer (0.1 M NaOH) [32]. The homogenates were resuspended in 2× Laemmli reducing-sample buffer and subjected to Western blot analysis.

### F-Actin Protein Binding Assay

To test the ability of Capzb2Δ106–140 to bind to actin an Actin Binding Protein, Biochem Kit (Cytoskeleton # BK013) was used according to the manufacturer's instructions. Briefly, all test proteins (including controls) were initially centrifuged at 150,000 *g* for 1 h at 4°C to remove the insoluble debris. Nonmuscle actin (23 µM) was polymerized for 10 min to form F-actin. F-actin (23 µM) was incubated with each of the following proteins (30 min, room temperature [RT]): α-actinin (positive control, as per the manufacturer's instruction), BSA (negative control, as per the manufacturer's instruction), Capzb2 (20 µM, as suggested by the manufacturer), and Capzb2Δ106–140 (20 µM). The incubates were centrifuged at 150,000 *g* (90 min, RT). The supernatant from each tube was removed and supplemented with Laemmli reducing sample buffer. The pellet from each tube was resuspended in cold deionized water, and Laemmli reducing-sample buffer was added. Equal amounts of both the supernatant and pellets from each reaction were loaded onto a 12% polyacrylamide gel and subjected to SDS-PAGE. Following electrophoresis, the gels were stained with Coomassie Brilliant Blue stain (Bio-Rad). Densitometry was performed to determine the percentage of each protein that bound to F-actin.

### Statistical Analysis

Statistical analysis (Graph Pad Prism version 4) was performed on each dataset using the following tests: paired (Figure 5I) and unpaired (Figure 3C) two-tailed Student *t*-test for single comparisons and one-way ANOVA with subsequent Tukey honestly significant difference test for multiple comparisons (Figures 2A, 2F, 6B, 6C, and Figure S2B). All statistical tests were performed at a 99% confidence interval.

## Supporting Information

Figure S1 **Capzb2 shRNA construct efficiently knocks down the expression of Capzb2 in CAD cells.** CAD cells were transfected with either control shRNA or Capzb2 shRNA. Lysates were prepared 48 h posttransfection and analyzed by Western blot using Capzb2 antibody.
(0.08 MB TIF)

Figure S2 **Capzb2 is expressed in the developing and adult mouse brain.** (A) Although the adult cortical Capzb2 expression is diminished in comparison to developmental levels (E16), the expression of Capzb2 in the hippocampus remains high in adulthood (representative Western blot). (B) Relative Capzb2 levels in the developing (E16) cortex, adult cortex, and adult hippocampus in comparison to developing (E16) hippocampus. Relative Capzb2 levels in each structure and time point are expressed as a mean of the ratio between Capzb2 and GAPDH densitometry from multiple Western blots (*n* = 11, mean value±the standard error of the mean [s.e.m.] are depicted; *** = *p*<0.001); values were normalized to the E16 hippocampus value (100%). Note that in the adult brain, Capzb2 levels in the hippocampus are significantly higher than in the cortex; the developing cortex contains significantly higher levels of Capzb2 than the adult cortex.
(0.36 MB TIF)

Figure S3 **Purified GST-tagged proteins (2 µg), fractionated and stained upon SDS-PAGE, used in tubulin polymerization assays, in vitro binding assays, and pull-downs from mouse brain lysates.** The identity of the proteins was also confirmed by Western blot with Capzb2 and/or GST antibody (unpublished data).
(0.69 MB TIF)

Figure S4 **The expression of EGFP-tagged RNAi-resistant Capzb2 full length and Capzb2Δ106–140 in CAD cells.** Note that the levels of RNAi-resistant Capzb2-EGFP (protein used in experiments depicted in Figure 2A and 2F) and EGFP-Capzb2Δ106–140 (protein used in experiments depicted in Figure 6) are comparable.
(0.09 MB TIF)

Figure S5 **Controls for Capzb2Δ106–140 and Capzb2 bindings of F-actin.** α-Actinin (10 µM) and BSA (51 µM) were incubated alone or in the presence of F-actin (23 µM). Upon centrifugation, the supernatant (S) and pellet (P) of each reaction were analyzed by SDS-PAGE followed by Coomassie Brilliant Blue staining. In the presence of F-actin, α-actinin cosediments with F-actin, whereas BSA remains in the supernatant.
(0.30 MB TIF)

## Abbreviations

C-zone: central zone
DIC: differential interference contrast
P-zone: peripheral zone
RNAi: RNA interference
ROI: region of interest
shRNA: small hairpin RNA
T-zone: transitional zone
