# Supplementary figures and images for: Capzb2 Interacts with β-Tubulin to Regulate Growth Cone Morphology and Neurite Outgrowth
Source: PLoS Biol. 2009 Oct 6;7(10):e1000208. doi: 10.1371/journal.pbio.1000208 (PMC2748697; doi:10.1371/journal.pbio.1000208)

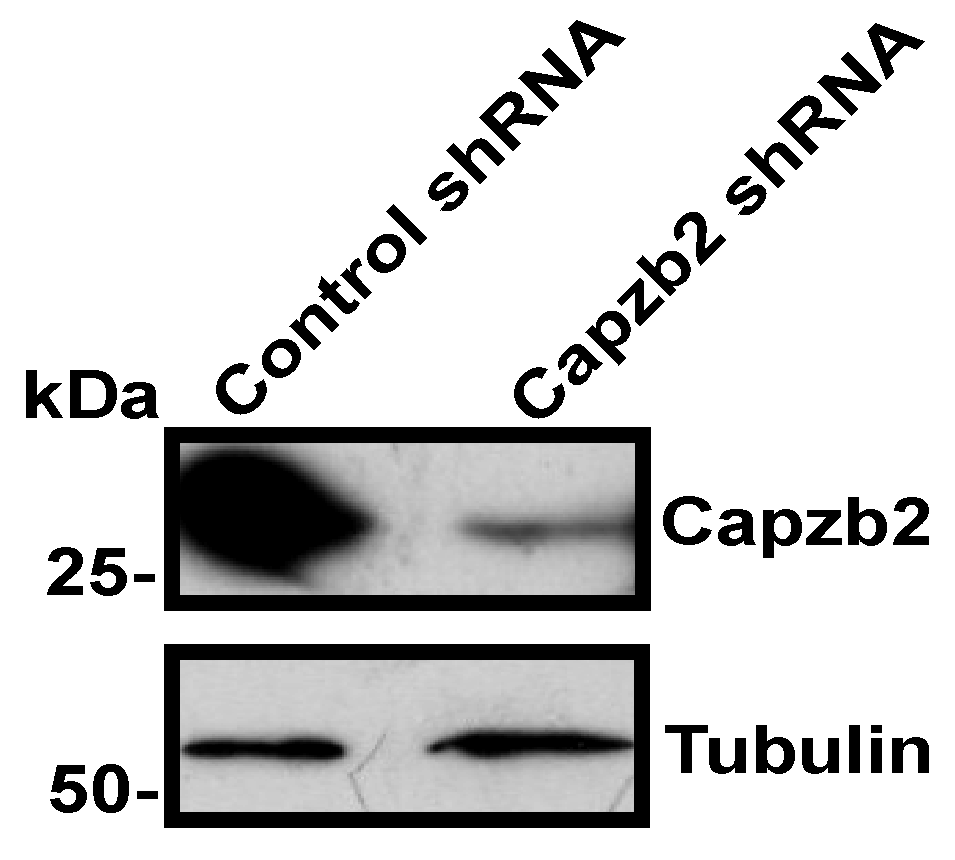

Supplement: Figure S1 — Capzb2 shRNA construct efficiently knocks down the expression of Capzb2 in CAD cells. CAD cells were transfected with either control shRNA or Capzb2 shRNA. Lysates were prepared 48 h posttransfection and analyzed by Western blot using Capzb2 antibody. (0.08 MB TIF) [file pbio.1000208.s001.tif]

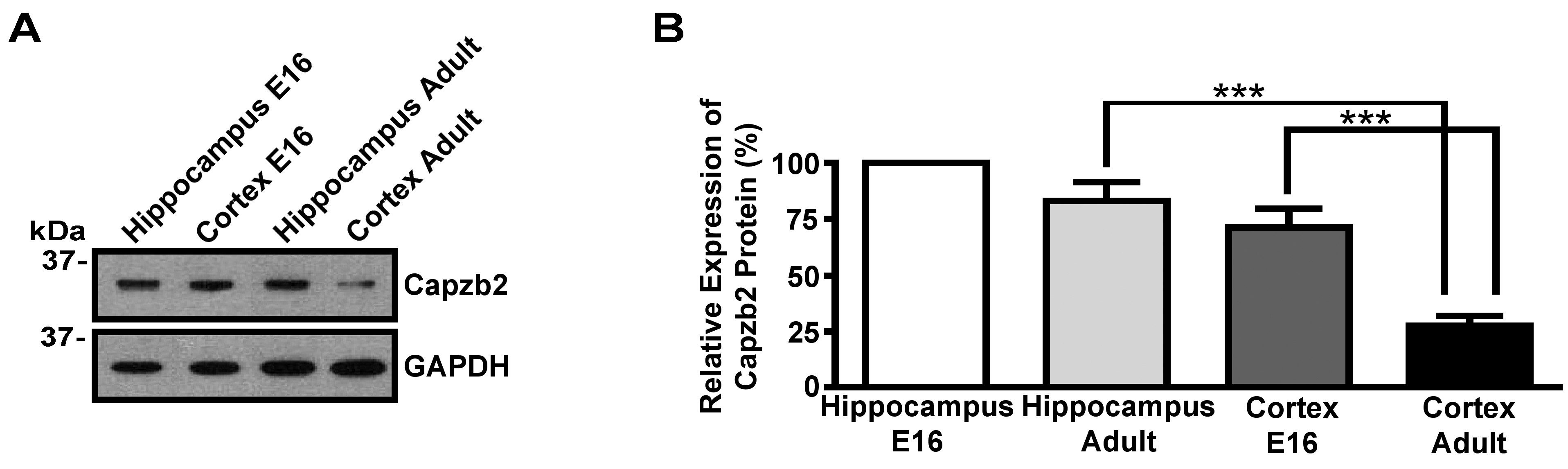

Supplement: Figure S2 — Capzb2 is expressed in the developing and adult mouse brain. (A) Although the adult cortical Capzb2 expression is diminished in comparison to developmental levels (E16), the expression of Capzb2 in the hippocampus remains high in adulthood (representative Western blot). (B) Relative Capzb2 levels in the developing (E16) cortex, adult cortex, and adult hippocampus in comparison to developing (E16) hippocampus. Relative Capzb2 levels in each structure and time point are expressed as a mean of the ratio between Capzb2 and GAPDH densitometry from multiple Western blots (n = 11, mean value±the standard error of the mean [s.e.m.] are depicted; *** = p<0.001); values were normalized to the E16 hippocampus value (100%). Note that in the adult brain, Capzb2 levels in the hippocampus are significantly higher than in the cortex; the developing cortex contains significantly higher levels of Capzb2 than the adult cortex. (0.36 MB TIF) [file pbio.1000208.s002.tif]

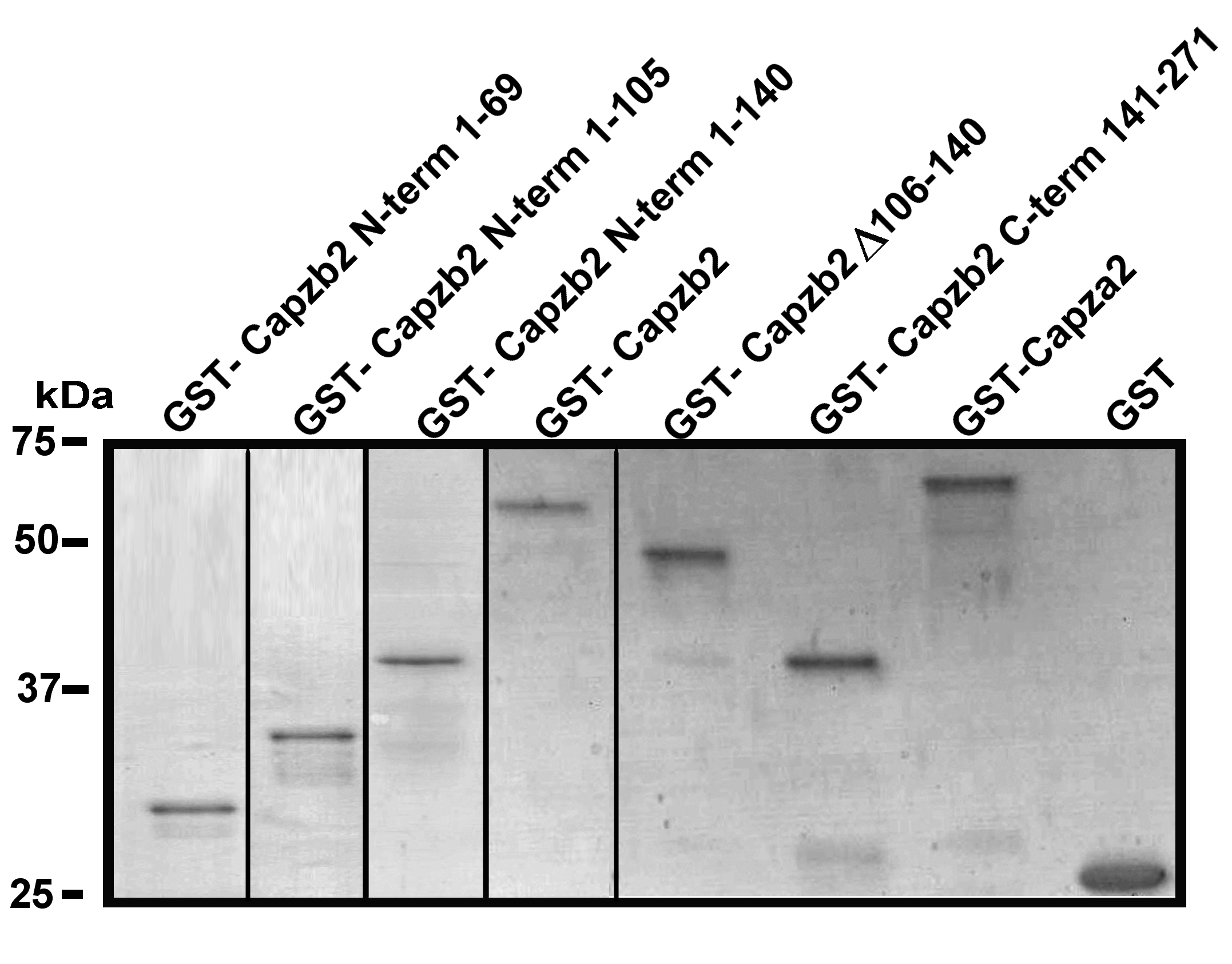

Supplement: Figure S3 — Purified GST-tagged proteins (2 µg), fractionated and stained upon SDS-PAGE, used in tubulin polymerization assays, in vitro binding assays, and pull-downs from mouse brain lysates. The identity of the proteins was also confirmed by Western blot with Capzb2 and/or GST antibody (unpublished data). (0.69 MB TIF) [file pbio.1000208.s003.tif]

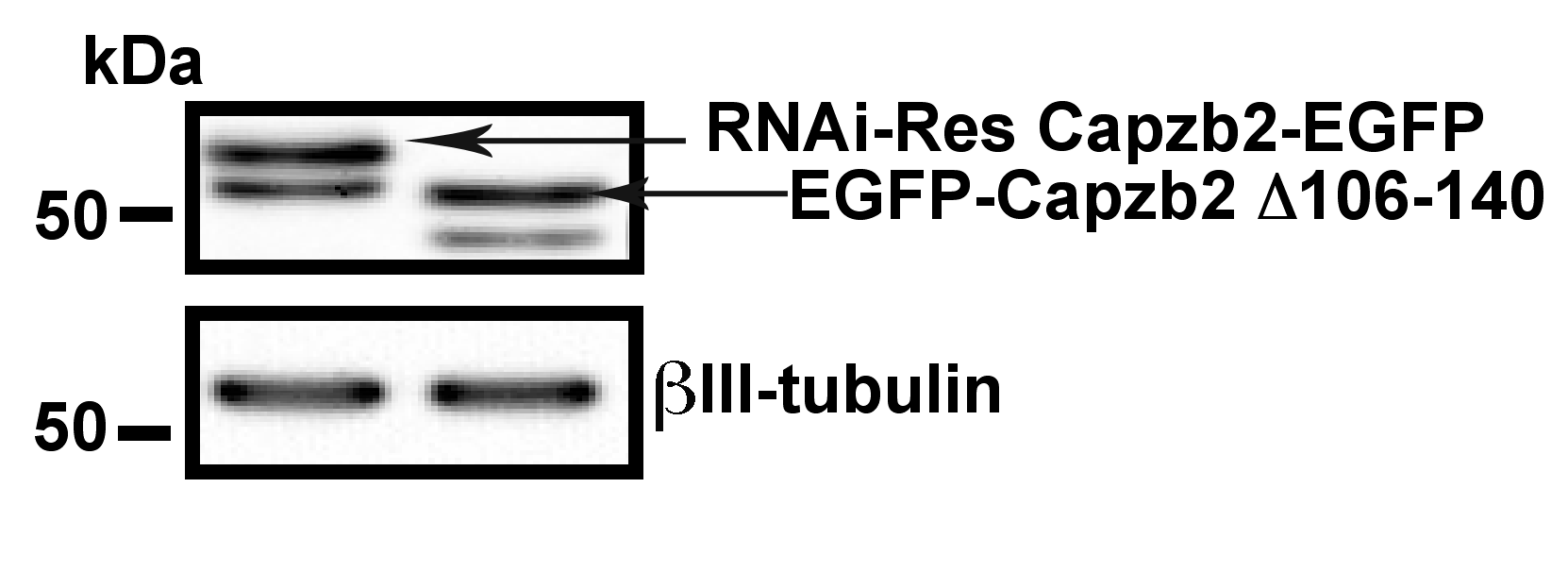

Supplement: Figure S4 — The expression of EGFP-tagged RNAi-resistant Capzb2 full length and Capzb2Δ106–140 in CAD cells. Note that the levels of RNAi-resistant Capzb2-EGFP (protein used in experiments depicted in Figure 2A and 2F) and EGFP-Capzb2Δ106–140 (protein used in experiments depicted in Figure 6) are comparable. (0.09 MB TIF) [file pbio.1000208.s004.tif]

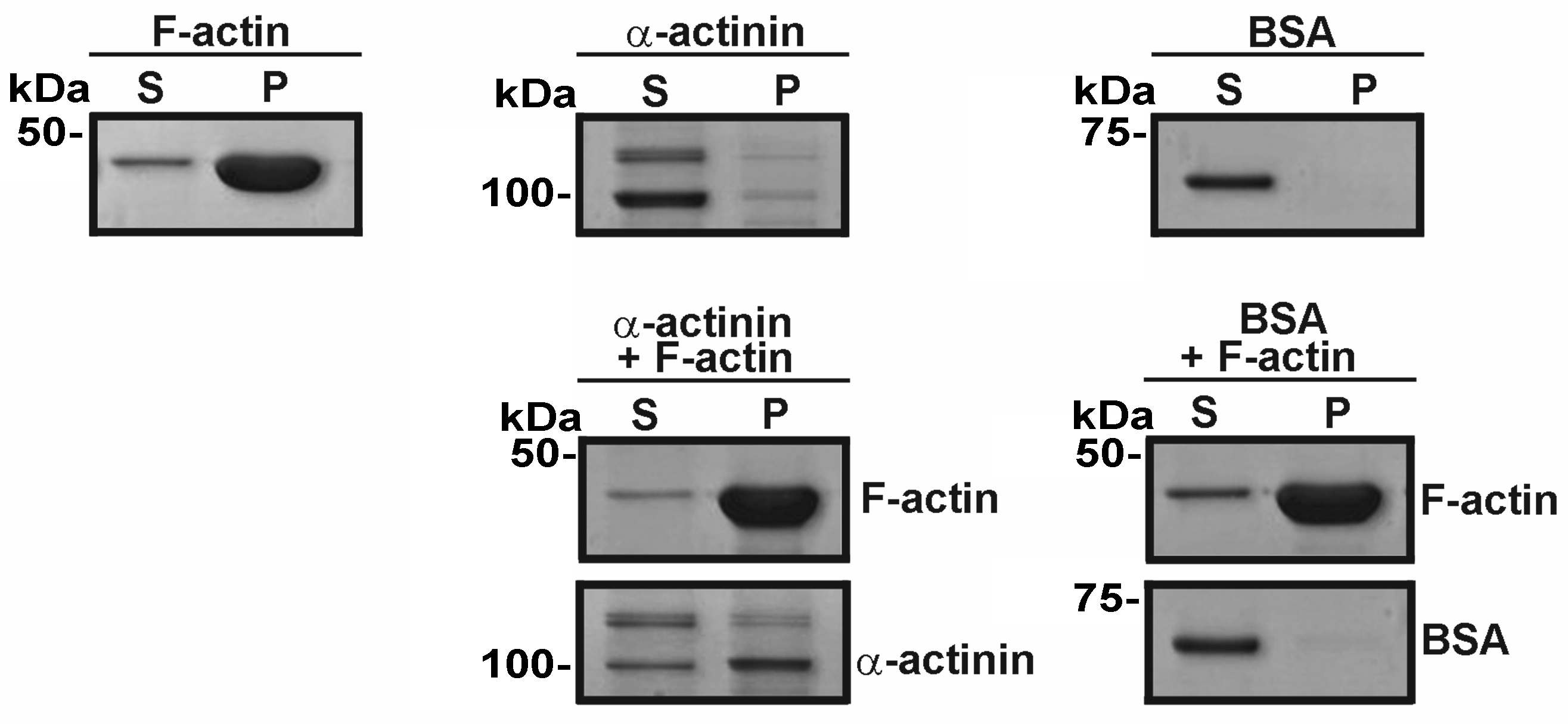

Supplement: Figure S5 — Controls for Capzb2Δ106–140 and Capzb2 bindings of F-actin. α-Actinin (10 µM) and BSA (51 µM) were incubated alone or in the presence of F-actin (23 µM). Upon centrifugation, the supernatant (S) and pellet (P) of each reaction were analyzed by SDS-PAGE followed by Coomassie Brilliant Blue staining. In the presence of F-actin, α-actinin cosediments with F-actin, whereas BSA remains in the supernatant. (0.30 MB TIF) [file pbio.1000208.s005.tif]
